# The Use of Digital Devices in the Management of Athletes with Paroxysmal Arrhythmias During Exercise—A Case Series

**DOI:** 10.3390/jcm15062170

**Published:** 2026-03-12

**Authors:** Mariusz Kłopotowski, Paweł Derejko, Łukasz Małek

**Affiliations:** 1 Department of Interventional Cardiology and Angiology, National Institute of Cardiology, 04-628 Warsaw, Poland; mklopotowski@ikard.pl; 2 Department of Cardiology, Medicover Hospital, 02-972 Warsaw, Poland; pderejko@yahoo.com; 3 Department of Cardiac Arrhythmias, National Institute of Cardiology, 04-628 Warsaw, Poland; 4 Department of Nursing, Faculty of Rehabilitation, University of Physical Education in Warsaw, 00-968 Warsaw, Poland

**Keywords:** wearable devices, athletes, supraventricular tachycardia, atrial fibrillation, diagnosis

## Abstract

**Background**: Athletes may experience paroxysmal arrhythmias that occur during exercise and are difficult to document using standard diagnostic modalities. Such arrhythmias are often unpredictable, transient, and cannot be reproduced during routine exercise testing or ambulatory electrocardiographic monitoring, leading to prolonged diagnostic pathways and uncertainty regarding management. **Methods**: This case series presents ten athletes in whom clinically relevant paroxysmal arrhythmias were initially detected using commercially available wearable digital devices, primarily chest-strap heart rate monitors and smartwatches. **Results**: In most cases, arrhythmias could not be documented using conventional diagnostic methods despite repeated investigations. Most presented athletes were referred for invasive electrophysiological study, which confirmed supraventricular arrhythmias and enabled curative catheter ablation based solely on data obtained from wearable devices. The use of digital devices substantially shortened the time to diagnosis and treatment, reduced diagnostic burden, and allowed definitive therapy in symptomatic athletes. **Conclusions**: Wearable technology, particularly chest-strap heart rate monitors, may play an important role in the diagnostic evaluation of exercise-induced paroxysmal arrhythmias when standard methods fail.

## 1. Introduction

Athletes, similarly to the general population, may experience paroxysmal arrhythmias (PA) [1,2]. In some individuals, these arrhythmias are triggered by physical exertion and occur only under specific circumstances, such as competition, high-intensity interval training, or prolonged endurance exercise. Their onset is often unpredictable, yet they may significantly impair performance, disrupt training plans, or necessitate termination of exercise.

Some paroxysmal arrhythmias are associated with pronounced symptoms, including palpitations, loss of power, dizziness, or presyncope, whereas others primarily affect athletic performance without causing overt haemodynamic compromise. Even when self-terminating, these episodes frequently generate anxiety related to the possibility of life-threatening cardiac events [1,2].

By their nature, paroxysmal arrhythmias often cease before they can be documented by medical personnel. The majority are supraventricular and occur in the absence of structural heart disease [1,2]. Consequently, establishing a definitive diagnosis may be challenging. Many athletes undergo repeated ambulatory ECG monitoring, exercise testing, or prolonged monitoring using patches or external/implantable loop recorders, frequently without success. In selected cases, invasive electrophysiological study (EPS) is required to confirm the diagnosis [3,4]. Even then due to the parasympathetic predominance, it is generally difficult to observe or trigger arrhythmias in baseline conditions as conduction through the atrioventricular junction is “slowed.” After administration of a beta-agonist, conduction improves, which improves the induction of the arrhythmia.

Accurate identification of the arrhythmia is essential for appropriate risk stratification and management. In athletes without structural or inherited heart disease, recurrent symptomatic supraventricular arrhythmias are commonly an indication for catheter ablation, which may provide definitive cure and enable safe return to sport [3,4].

Recent years have seen the rapid development of wearable heart rate and ECG monitoring technologies, including chest straps and smartwatches [5,6,7]. These devices offer continuous monitoring during training and competition and may facilitate documentation of otherwise elusive arrhythmias. In this case series, we present ten athletes of different ages and sporting disciplines whose paroxysmal arrhythmias were initially detected using wearable devices and who were referred for treatment mainly based solely on this form of arrhythmia documentation.

## 2. Case Series

All presented consecutive athletes were evaluated in the private practice of an experienced sports cardiologist between 2021 and 2025. Each athlete sought consultation because of palpitations occurring during exercise, characterized by an abrupt increase in heart rate disproportionate to exercise intensity and loss of power, dizziness or presyncope. During these episodes, most athletes were forced to interrupt training or continue at markedly reduced intensity. In all cases, the arrhythmia terminated spontaneously either during or shortly after exercise.

The frequency of episodes ranged from weekly to once every several months, and onset was consistently unpredictable. Arrythmia was present for months to up to 2 years, with some athletes already seeking medical advice without success. All athletes were either professional or semi-professional and represented various sporting disciplines. Importantly, they were highly familiar with their physiological heart rate responses to exercise. 

All patients underwent resting 12-lead ECG, transthoracic echocardiography, maximal exercise testing, and between one and several 24 to 72 h ambulatory ECG recordings. At baseline, all athletes were free of known heart disease, otherwise asymptomatic, and in excellent physical condition.

**Figure 1:**
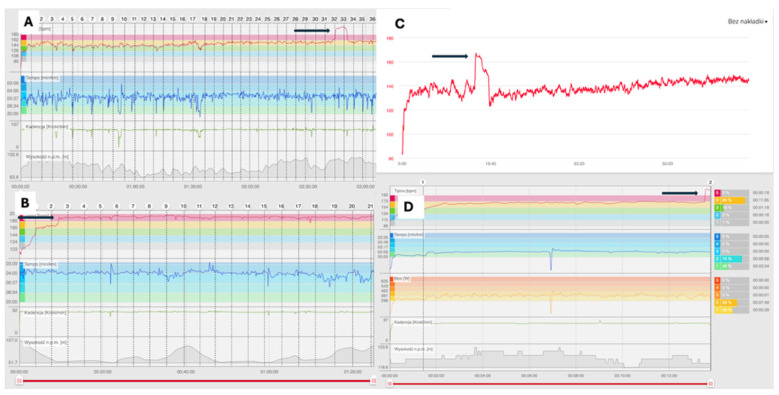
Examples of heart rate (HR) tracing from the chest strap demonstrating paroxysmal tachycardia. (**A**) The trace from a 39-year-old male ambitious runner (personal best (PB) in a half-marathon 1:09), showing paroxysm of arrythmia after 2 h and 30 min of a training long-run (black arrow) with a sudden HR increase to 205 beats per minute (bpm), which ceased spontaneously despite continued exercise. (**B**) The same athlete with arrythmia onset after 3 km of a half-marathon (black arrow). The arrhythmia persisted until the end of the race but caused significant decrease in running pace by the end. (**C**) The HR trace of a 68-year-old triathlonist, showing an irregular heart rate increase during cycling training (black arrow). (**D**) The trace of a 30-year-old elite triathlonist (PB in full ironman < 8 h) with arrythmia onset after 12 min of training (black arrow) to over 210 bpm, which forced the termination of training. A similar paroxysm was observed by the athlete during swimming, causing dizziness and anxiety.

**Figure 2:**
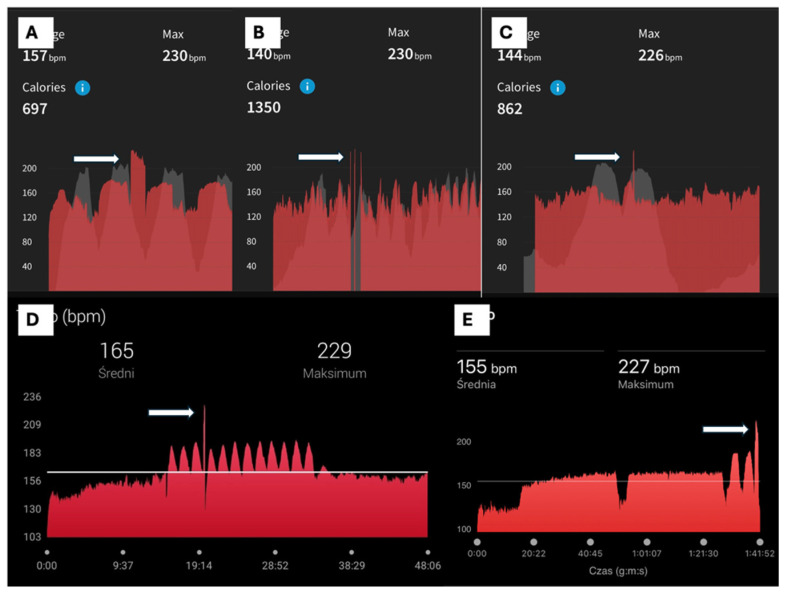
Examples of heart rate (HR) tracing from the chest strap, demonstrating paroxysmal tachycardia. (**A**–**C**) A 21-year-old male professional MTB cyclist with longer paroxysms and short runs of arrhythmia with HR up to 230 bpm (white arrows). (**D**,**E**) An ambitious 30-year-old female runner with a short paroxysm of arrhythmia during interval training increasing the HR to almost 230 bpm (white arrows).

In each case, the athletes identified a characteristic, abrupt, and reproducible heart rate pattern on their wearable devices that coincided with symptoms and differed clearly from motion artefacts (Figure 1 and Figure 2).

Patient characteristics are summarized in Table 1.

**Figure 3:**
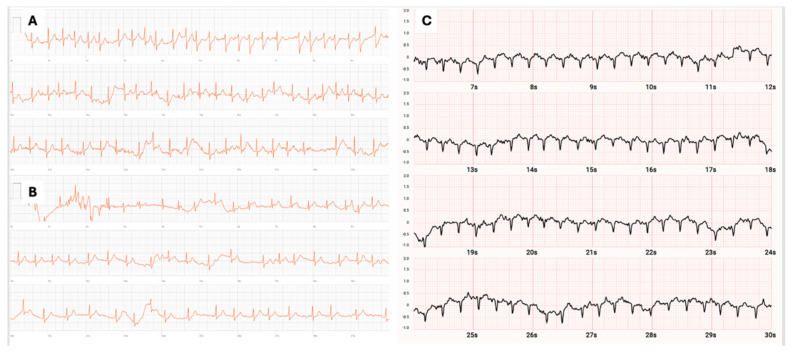
Two traces from smartwatch one-lead ECG demonstrating supraventricular tachycardia. (**A**,**B**) A 46-year-old semi-professional cyclist with a heart rate of 112 bpm during an episode of atrial fibrillation (AF) recorded during a pause in training. Some artefacts are visible due to the demanding conditions of the arrhythmia registration. (**C**) Arrhythmia in an elite triathlonist presented in Figure 1D, showing regular supraventricular tachycardia 210 bpm diagnosed during EPS as atrioventricular nodal re-entrant tachycardia (AVNRT).

Maximal heart rate during arrhythmic episodes ranged from 205 to 240 bpm, clearly exceeding age-predicted maximal heart rates. Most arrhythmias were detected exclusively using chest-strap heart rate (HR) monitors. In one case, wrist-based photoplethysmography (PPG) was used, and in two cases the heart rate increase was supported by single-lead ECG recordings from smartwatches (Figure 3).

Only four athletes (40%) had arrhythmias documented using standard diagnostic methods such as ambulatory ECG or exercise testing. In one individual, several ambulatory ECG recordings combined with a prolonged, near-maximal exercise protocol were required to capture the arrhythmia.

Structural heart disease was excluded in all but one athlete, a 48-year-old cyclist with a bicuspid aortic valve and moderate aortic stenosis. This athlete declined EPS and remains under close echocardiographic surveillance.

In our group, all athletes with justified presence of the symptomatic isolated supraventricular arrythmia based on arrythmia analysis from a wearable device and willing to undergo ablation to eliminate the arrhythmia background were referred for EPS. Time from wearable detection to EPS was between 6 months and 2.5 years. In all cases, EPS successfully induced supraventricular arrhythmias, including atrial fibrillation (n = 4), atrioventricular nodal re-entrant tachycardia (AVNRT; n = 3), and atrioventricular re-entrant tachycardia due to accessory pathway (AVRT; n = 2). All patients underwent catheter ablation during the same procedure. One athlete with AVRT required a repeat ablation due to the presence of a second accessory pathway.

All athletes gradually returned to training and competition and remained free from symptoms during follow-up ranging from 1 to 4 years. Additionally, 24 h ambulatory ECG recordings with exercise after 3–6 months from ablation did not disclose the presence of arrythmia.

## 3. Discussion

Current Heart Rhythm Society and European Heart Rhythm Association recommendations acknowledge the role of wearable digital devices in athletes who experience infrequent or difficult-to-document palpitations [2,5]. Commercially available devices, including fitness trackers, smartwatches, handheld electrodes, and chest-strap ECG monitors, allow rhythm data to be stored and shared with healthcare professionals. Despite these recommendations, published data demonstrating their use remain limited and show variable accuracy [8].

In this case series, we demonstrate the clinical utility of wearable devices in a cohort of advanced endurance athletes of both sexes, including both young and master athletes. In all but one individual, a definitive diagnosis confirmed by EPS was prompted by data obtained from wearable devices. In several cases, these data constituted the only objective evidence of arrhythmia yet were considered sufficient to justify invasive evaluation, which ultimately led to curative ablation.

The use of wearable devices substantially shortened the diagnostic pathway, reduced healthcare utilization, and alleviated anxiety among athletes. In well-trained individuals who can distinguish artefacts from true arrhythmic events, these devices appear particularly valuable. Chest-strap heart rate monitors proved especially useful for detecting mid-exercise arrhythmias, which were often missed by smartwatch-based PPG or ECG recordings [8,9].

Smartwatch PPG is known to be less reliable during intense exercise, whereas smartwatch ECG requires a stable position and a minimum recording duration, conditions that are often incompatible with high-intensity physical activity [5]. Consequently, some arrhythmic episodes were too brief or occurred under conditions unsuitable for ECG recording [8].

Recordings obtained during exercise are particularly prone to motion artefacts, signal noise, and algorithm misclassification, especially in the setting of rapid sinus tachycardia. Therefore rhythm assessment based on heart rate-only tracings without systematic ECG validation during exercise should be interpreted with caution. Characteristic features of paroxysmal arrhythmias detected using heart rate monitoring, which may help in decision-making towards EPS referral, included

1. Sudden and atypical heart rate increase exceeding expected maximal values;
2. Concomitant symptoms such as palpitations, loss of power, dizziness, or presyncope;
3. Reproducibility across different training sessions;
4. Varied duration, ranging from seconds to over an hour;
5. Termination with cessation of exercise.

While ECG documentation remains essential for precise arrhythmia classification, single-lead ECG is not always sufficient. When non-invasive methods fail, invasive EPS with provocation remains the definitive diagnostic tool and allows immediate treatment [2,3,4].

Consistent with previous reports, younger athletes in our series were more likely to present with AVNRT or AVRT, whereas atrial fibrillation (AF) predominated among master athletes, a pattern that may aid differential diagnosis in clinical practice [10,11]. Importantly, AF in athletes is often vagally mediated. In these circumstances, AF typically occurs under conditions of pronounced vagal predominance, most commonly during nighttime or rest. Nevertheless, AF can also be adrenergic (exercise-induced) and may start during exercise and terminate shortly after cessation [1].

## 4. Conclusions

This case series supports the use of wearable digital devices, particularly chest-strap heart rate monitors, in the detection of exercise-induced paroxysmal arrhythmias in athletes. When characteristic features are present, data obtained from these devices may support referral for an invasive electrophysiological evaluation, even in the absence of arrhythmia documentation using standard methods. Our findings contribute to the growing body of evidence supporting broader integration of wearable technologies into the diagnostic pathways of sports cardiology.

## Figures and Tables

**Table 1:** Patient characteristics.

Case	Age	Sex	Sport	Maximal HR During Arrhythmia	Digital Device Used to Detect Arrythmia	Ambulatory ECG Monitoring, Exercise Test Repeatability	EPS Diagnosis
Athlete 1	30	Male	Triathlon	211	Polar chest strap + Polar watch ECG	0	AVNRT
Athlete 2	30	Female	Running	229	Garmin chest strap	0	AVRT
Athlete 3	21	Female	MTB	230	Garmin chest strap	0	AVNRT
Athlete 4	39	Male	Running	205	Polar chest strap	0	AVNRT
Athlete 5	33	Male	Windsurfing	240	Garmin chest strap	Ambulatory ECG and exercise test	AVRT
Athlete 6	18	Female	Handball	230	Polar chest strap	Ambulatory ECG	AVNRT
Athlete 7	68	Male	Triathlon	220	Garmin watch PPG	Exercise test on a treadmill	AF
Athlete 8	48	Male	Cycling	200	Garmin chest strap	0	-
Athlete 9	36	Male	Running	230	Suunto chest strap	After several ambulatory ECGs, exercise test on a treadmill with a long exercise test protocol	AF
Athlete 10	46	Male	Cycling	215	Samsung watch ECG	0	AF

AF—atrial fibrillation; AVNRT—atrioventricular nodal re-entrant tachycardia; AVRT—atrioventricular re-entrant tachycardia; ECG—electrocardiogram; EPS—electrophysiologic study; HR—heart rate; MTB—mountain-bike.

## Data Availability

Data are available from the authors.

## Abbreviations

The following abbreviations are used in this manuscript:

ACC	American College of Cardiology
AF	Atrial fibrillation
AHA	American Heart Association
AVNRT	Atrioventricular nodal re-entrant tachycardia
AVRT	Atrioventricular re-entrant tachycardia
bpm	Beats per minute
EAPC	European Association of Preventive Cardiology
ECG	Electrocardiogram
EHRA	European Heart Rhythm Association
ESC	European Society of Cardiology
EPS	Electrophysiological study
HR	Heart rate
HRS	Heart Rhythm Society
MTB	Mountain bike
PA	Paroxysmal arrhythmia
PB	Personal best
PPG	Photoplethysmography
